# Photooxidative Behavior of Polystyrene Nanocomposites Filled with Two-Dimensional Molybdenum Disulfide

**DOI:** 10.3390/polym15092099

**Published:** 2023-04-28

**Authors:** Aurianny Lima Angulo, Camila Laura Celis Rodriguez, Guilhermino José Macedo Fechine

**Affiliations:** 1 School of Engineering, Mackenzie Presbyterian University, Rua da Consolação, 930—Consolação, São Paulo 01302-907, Brazil; annyauri@gmail.com (A.L.A.); celis.camilalaura@gmail.com (C.L.C.R.); 2 MackGraphe—Mackenzie Institute for Research in Graphene and Nanotechnologies, Mackenzie Presbyterian Institute, Rua da Consolação, 930—Consolação, São Paulo 01302-907, Brazil

**Keywords:** photodegradation, two-dimensional filler, nanocomposites, polystyrene, molybdenum disulfide

## Abstract

This study aimed to investigate how an ultralow content of a molybdenum disulfide (MoS_2_) two-dimensional particle affects the photodegradation mechanism of polystyrene (PS). Here, an accelerated weathering study was presented on neat polystyrene and its nanocomposites produced with 0.001, 0.002, 0.003 and 0.005 wt% of molybdenum disulfide (MoS_2_) exposed for various irradiation intervals (up to 8 weeks). The polymer photo-transformations were monitored using size exclusion chromatography (SEC), infrared spectroscopy (FTIR), and UV-Vis spectroscopy. The FTIR and UV/Vis results indicate that the PS degradation mechanism was not altered by the presence of MoS_2_ particles; however, the degradation reactions were slowed down at higher MoS_2_ contents (>0.003%). The SEC results proved the stabilizer effect due to MoS_2_ particles, where ${\bar{M}}_{n}$, ${\bar{M}}_{w}$, and ${\bar{M}}_{w}$/${\bar{M}}_{n}$ values after 8 weeks were less modified when compared with the neat PS results. The MoS_2_ acted as a UV stabilizer, and these two-dimensional particles acted by deactivating the free radicals generated by the PS matrix, even considering the low amount of the filler (<0.005 wt%).

## 1. Introduction

Since the isolation of graphene, materials with a lamellar structure were proven to be quite attractive due to their distinct mechanical, thermal, and electrical characteristics [1,2]. Similarly, the inorganic layered molybdenum disulfide (MoS_2_), a typical representative of the transition metal dichalcogenide family, is built up of a Mo plane sandwiched by two planes of S held together by covalent bonds, and S-Mo-S units bonded by van der Waals forces [3,4]. As the bulk form is reduced to the nanoscale, the physical, optical, and electronic characteristics differ due to the change in energy levels and electron availability; the unexfoliated material has a bandgap of 1.20 eV, while the 2D reaches a gap of 1.90 eV. Naturally, such properties vary with the number of layers [3,5]. In terms of applicability, the two-dimensional material expands and diversifies the possibilities of the conventional dry lubricant, established in the industry [6,7,8,9], for use in sensors [5], biomedicine [10], electronics [11], environmental correction [12], hydrogen production [13], and photocatalysis [14].

The unique photochemical reactivity of MoS_2_ turns this 2D material into an emerging and promising player in the photooxidative process due to its capacity for generating or scavenging highly reactive species [15,16]. In contexts where the nanoparticle is relevant in the degradation of organic pollutants via photocatalytic oxidation, UV irradiation may cause the formation of reactive oxygen species [13,14,17]. These species are observed in polymeric systems under distinct environments and aging conditions. They are also typical in pro-oxidant fillers since they are implicated in various important photo-transformation processes [15,18,19,20]. On the other hand, under physiological conditions, the MoS_2_ nanosheets were already reported as a free radical quencher and scavenger [21,22,23]. The MoS_2_ UV radiation performance was shown to be related to its crystalline structures, particle concentration, and level of layered structures [21,22,23,24].

Specifically, the layered nanoparticles were used as fillers for polymeric matrices [25,26,27,28,29]. The unique properties of these two-dimensional materials (2DMs) are very attractive and lead to the production of high-performance nanocomposites. Nanocomposites with conventional fillers generally have a much higher filler content when compared to nanocomposites with two-dimensional materials as fillers. This is because very low levels of two-dimensional materials are needed to positively affect the properties of the polymers (mechanical, thermal, electrical, and others) due to their higher aspect ratio and enormous surface area, mainly for 2DMs with few layers [30,31,32,33].

The results of Rodriguez et al. recently showed that very low contents of MoS_2_ (0.001–0.005 wt%) added to polystyrene (PS) improved the mechanical properties. When 0.002 wt% of MoS_2_ is added to PS, tensile strength values increase by 18%, elongation at break increases by 31%, toughness increases by 57%, and Young’s modulus showed a small decrease in PS stiffness, around 11% [34]. In respect to thermal stability, the thermogravimetric analysis (TGA) highlighted that the presence of MoS_2_ particles induces an increase around 10 °C in the initial weight loss temperature, despite the temperatures corresponding to the advance of the mass loss process not changing. Although the compositions cannot be associated with a better thermal stability performance, it is noteworthy that the results indicate a delay in the thermal degradation of the nanocomposites when compared to the pure material, which may be the result of a better heat dissipation between the polymeric matrix and the MoS_2_.

This result is even more significant when considering the incorporation of MoS_2_ in polymer matrix were made using melt-mixing. The melt-mixing strategy involved a specific control of processing parameters that allowed nanocomposites to be well-dispersed and distributed fillers in the matrix. As a twin-screw extruder was used, the process can be scaled up to industrial levels [35]. By combining the performance of these compositions with an appropriate processing method, the photo-oxidative behavior of such nanocomposites is extremely relevant, as durability is the key factor for possible future applications. These multifunctional materials have potential applications in many technological fields. The packaging industry, for example, currently demands transparent high-quality products.

**Scheme 1:**
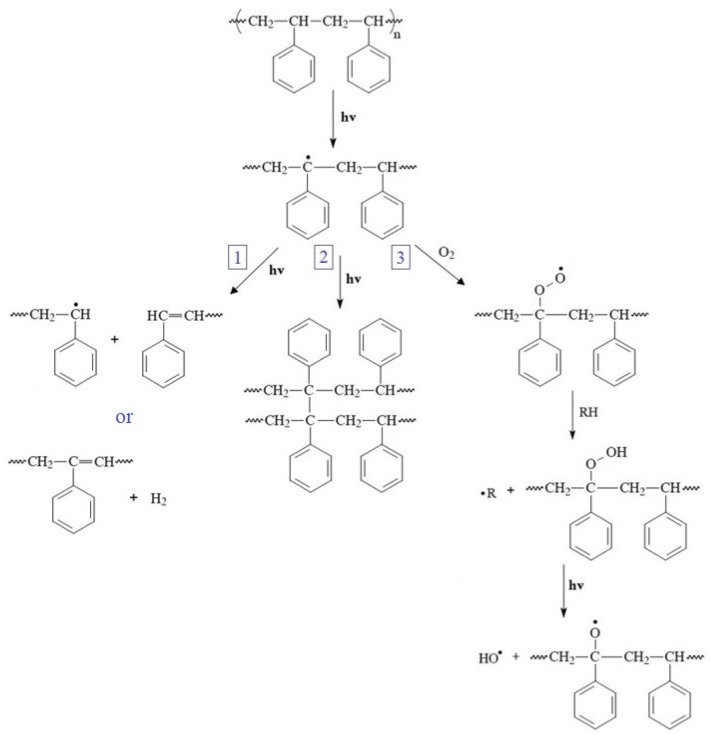
Photodegradation mechanism of polystyrene [37,38,39].

The PS degradation was extensively investigated, so the mechanisms and stabilization are well established. During the first stage of degradation (Scheme 1), the primarily created polymer alkyl radicals (P•) can interact using different pathways. Β scission may occur, forming end-chain saturation or conjugated double bonds along the polystyrene backbone (1) [36]. Cross-links reactions are also expected in the interaction between polystyryl radicals (2). In an oxygen environment, route (3) shows the formation of peroxyl radicals (POO•) and consecutively hydroperoxides (POOH). After the O-O dissociation, alkoxy (PO•), and hydroxyl radicals are formed, as shown in Scheme 1. The pathways of alkoxy interactions play a key role in the formation of most oxidation products [37]. One of the most known oxidation products is carbonyl groups, and they can be: ketone, aldehyde, carboxylic acid, ester, lactones, and hydroperoxides. Such structures are identified by FTIR spectroscopy. However, the interpretation of the occurrence of these functional groups is not simple, due to the overlap of different types of carbonyl species in the same frequency range. One way to qualify and quantify the occurrence of each of these oxygenated groups is by curve fitting of the carbonyl band in the FTIR spectrum. The filler addition as MoS_2_ may change the pathway degradation and it needs investigation.

In the case of nanocomposites, there is no published study about the effect of two-dimensional MoS_2_ on PS durability in terms of long-term ageing. In view of possible applications, the effect and how these two-dimensional fillers can interfere with the matrix photodegradation mechanism is of prime importance, since most polymer applications are submitted to UV radiation, the presence of oxygen, and, in some cases, humidity. It is well known that, in general, the stabilization additives of degradative processes entail high industrial costs [40]. In this regard, the results extracted from these new studies can guide the stabilization strategy of the final product, since the class and amount of the additives depends on how the MoS_2_ interferes with the PS degradation mechanism when exposed to UV radiation.

The present work aims to investigate, for the first time, the effect of very low content of molybdenum disulfide on the photodegradation process of polystyrene when exposed to controlled accelerated weathering. The samples used herein were obtained from the study of Rodrigues [34]. The films were analyzed by UV-vis spectroscopy, infrared spectroscopy (FTIR), and size exclusion chromatography (SEC). Qualitative and quantitative analysis of the chemical functions present in the surface of the photodegraded samples were performed using ATR-FTIR curve fitting.

## 2. Experimental Methods

### 2.1. Materials and Sample Preparation

The commercially available polystyrene (${\bar{M}}_{n}$ = 114,600 and ${\bar{M}}_{w}$ = 26,920) used was supplied by INNOVA. The molybdenum disulfide (MoS_2_), with 99% purity, in the 2H phase used in this work was provided by Sigma Aldrich. The two-dimensional particles had a lateral size of 0.1–0.2 μm with a 2.5–10.0 nm thickness, indicating ≤5 layers, and were obtained using liquid-phase exfoliation (LFE). The exfoliation procedures and characterization of the obtained MoS_2_ were already described in a previous work [34,41].

### 2.2. Production of PS/MoS_2_ Nanocomposites

The PS/MoS_2_ nanocomposites were prepared with a corotational twin-screw extruder L/D = 40 (process 11, Thermo Scientific) using the liquid phase feeding (LPF) technique previously described in the literature [35]. The method consists of adding a liquid dispersion of 2D material in a melted polymer matrix with a peristaltic pump positioned on an extruder at L/D = 10. The pump flow rate was controlled to produce nanocomposites with 0.001, 0.002, 0.003, and 0.005% wt% of MoS_2_. The compositions were pressed in a Solab uniaxial press at 230 °C for 3 min. The thickness values are within an average value equal to 350.

### 2.3. Accelerated Laboratory Weathering

The films were exposed to controlled accelerated weathering conditions in an industrial test chamber, Q-Lab. The irradiation source was composed of UVA fluorescent lamps, UVA-340. The weathering cycle was defined according to standard ASTM G-154, 8 h under UV light emission 0.89 W/m^2^ at 50 °C and 4 h in the dark under condensed water at 60 °C.

### 2.4. Characterization Techniques

#### 2.4.1. UV-Vis Spectroscopy

The absorption spectra in the ultraviolet region were measured using a Shimadzu spectrophotometer model UV-3600 Plus UV-Vis NIR operating in the range of 800–190 nm. The tests were performed directly on the films using an integration sphere coupled to the spectrophotometer.

#### 2.4.2. Fourier Transform Infrared Spectroscopy

The chemical characterization of neat PS and nanocomposites was carried out using an IR spectrophotometer with attenuated total reflectance (ATR) and IRAffinity (Shimadzu). The films were analyzed over the range of 4000–600 cm^−1^ at a resolution of 2 cm^−1^ with 120 scan repetitions. A separate background spectrum was subtracted for each collection.

The oxidation products were identified using mathematical curve fitting. The curve-fitting analysis of the range was performed using the OriginPro 2021 software package using the second derivative method. Prior to the mathematical analysis, the curves were normalized. The Gauss line shape and subtract baseline were used as the deconvolution strategy; this Gaussian profile provides the best fit. The fit quality is controlled using the coefficient R^2^ > 0.998 for the absorption band of each sample. The residual sum of the squares was determined to be chi-square χ^2^ < 10^−5^. The second order was potentially very useful for identifying hidden and overlapping peaks.

#### 2.4.3. Size Exclusion Chromatography

The molecular weights of the unexposed and irradiated nanocomposites were measured on a Malvern GPC-HT 350 A equipped with a refractive index detector. The column set consisted of a guard column and a combination of three separation columns (K-806M, Shodex). The calibration was performed using narrow distributed PS standards (PSS Polymer Standards Service GmbH). Chloroform was used as the eluent at a 1 mL/min flow rate at 40 °C. The sample volume of 100 μL was measured with a concentration of 1 mg/mL and was injected without previous filtration. ${\bar{M}}_{n}$, ${\bar{M}}_{w}$ and polydispersity index (${\bar{M}}_{w}/{\bar{M}}_{n}$) were determined using Viscotec (Malvern) software.

## 3. Results and Discussion

The observed visual appearance after irradiation ranged from translucent to yellow in the films with increasing exposure time. The images are displayed in Table 1. The conjugated double bounds along the polystyrene backbone generate polyene and are responsible for color change in the polystyrene photooxidation process [36].

### 3.1. UV-Vis Spectroscopy

**Figure 1:**
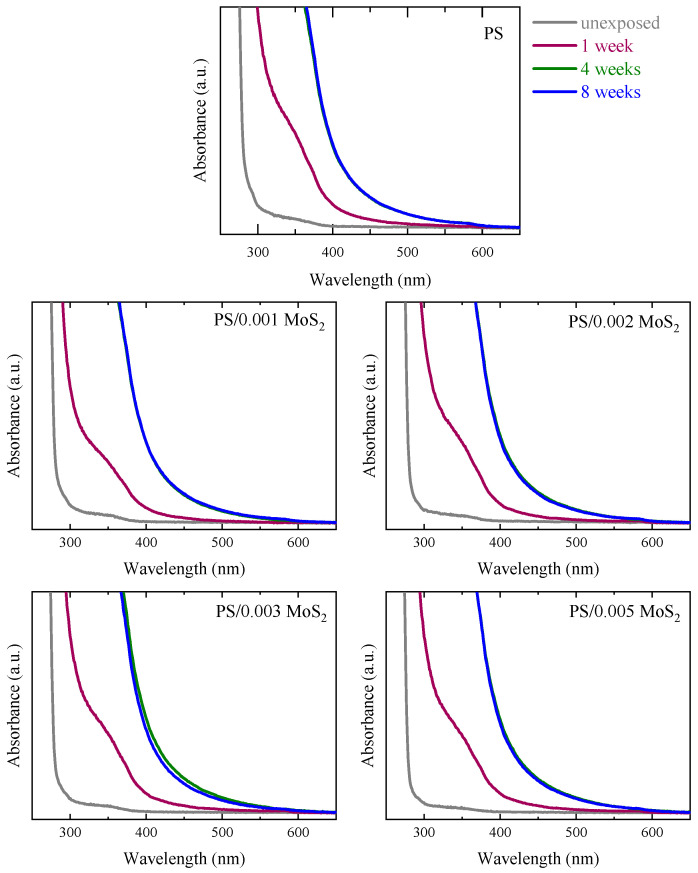
UV−Vis absorbance spectra of neat PS and PS/MoS_2_ nanocomposites exposed to UV radiation at different times.

From the spectroscopic analyses in the visible range, it is possible to identify the transitions in the absorption spectrum referring to the characteristics of chemical bonds. In this photodegradation context, such chemical bonds show the formation of carbonyl groups, aromatic rings, double bonds, and color change [39,42,43]. For neat polystyrene and all compositions, the absorbance increased in the range of λ = 280–500 nm (Figure 1). As the time of exposure to UV radiation increased, the absorbance increased intensely.

**Scheme 2:**
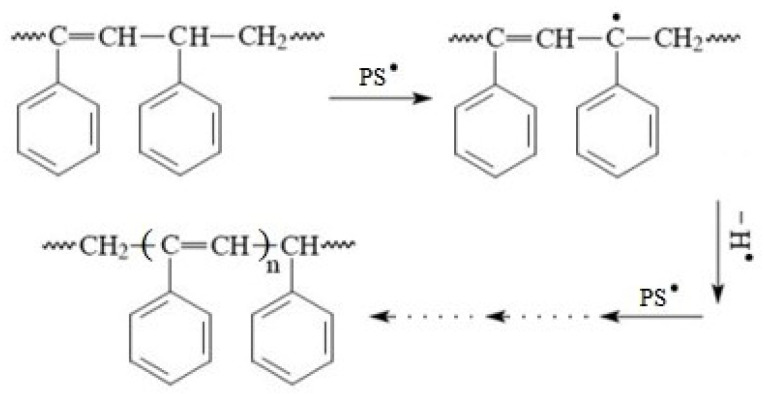
Polyene formation during photodegradation of polystyrene (dotted arrow: “n” reactions) [39,44].

The absorbance increase in this interval was due to the development of unsaturated bonds near carbonyl groups and conjugated double bonds. Species with aliphatic double bonds are represented in route 1 (Scheme 1). These alkenes were stable intermediates in the formation of polyenes with different numbers of double bonds, according to Scheme 2 [39,43]. The absorption of these species above λ = 300 nm contributed to the yellowing of the films and is typical in degradative processes of polystyrene under UV radiation [44,45].

**Figure 2:**
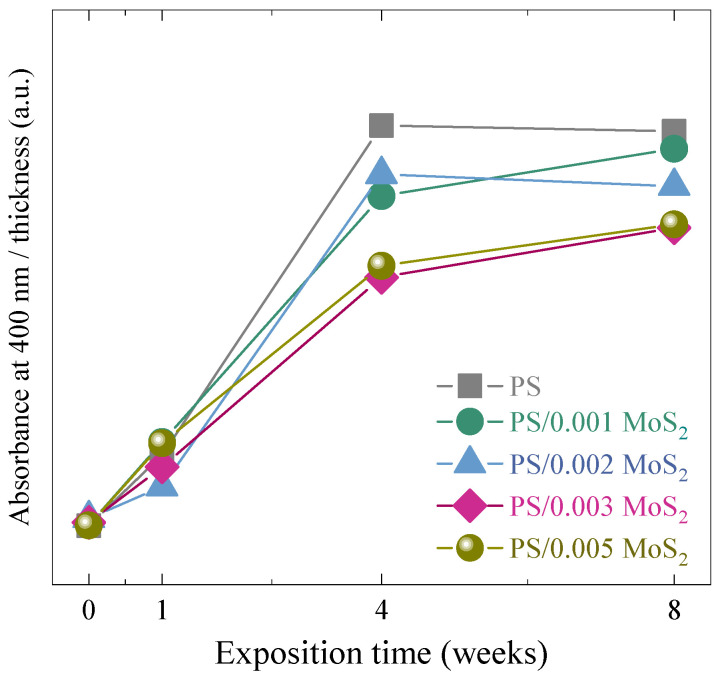
UV-Vis absorbance at 400 nm divided by sample thickness.

According to the Lambert–Beer law, the absorbance is proportional to the concentration of the molecules since the photons of the light beam will encounter a greater or lesser amount of absorbing chemical species when the light passes through a solution or film [46]. In this context, Figure 2 illustrates the relationship between absorbance/thickness as a function of the exposure time to accelerated aging, specifically considering the 400 nm wavelength. When considering the thickness in the analysis of the absorbance in the UV-Vis range, it can be observed that, for the period of 8 weeks, the absorbance was lower for the nanocomposites when compared to the neat PS values. This indicated less formation of unsaturated bonds near carbonyl groups and conjugated double bonds for the nanocomposites compared to PS. As observed in the photographic record, a low transmittance was observed in the PS film after the last irradiation period. This result may be indicative of the photoprotection power derived from MoS_2_ particles.

### 3.2. FTIR

**Figure 3:**
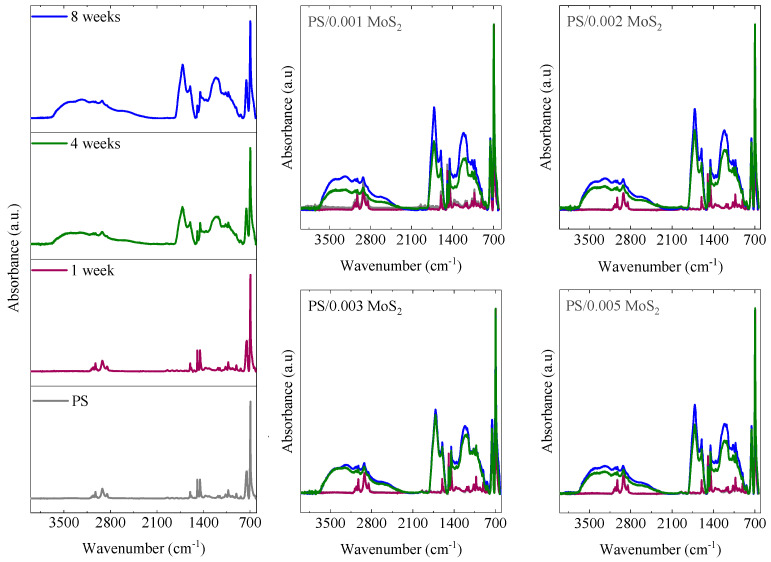
FTIR spectra of PS and PS/MoS_2_ nanocomposites exposed to UV radiation at different times.

The FTIR spectra for PS and nanocomposite samples, unexposed and exposed to UV radiation, are shown in Figure 3. The spectra measured before the weathering exposure shows the typical polystyrene bands.

Some regions of the spectrum showed changes in absorption intensities after weathering exposure, mainly after 4 weeks. A complex band at a frequency range of 3200–3600 cm^−1^ was related to hydroxy vibrations, typical of hydroperoxides, carboxylic acid, and alcohol. The alcohol resulted from the abstraction of a hydrogen atom from the polymeric chain by the alkoxy macroradical.

A broad band in the carbonyl region at about 1850–1650 cm^−1^ was also found in degraded samples. As mentioned in Scheme 1, the C=O function was the main chemical group generated during PS photooxidation 37. However, in this region, bands related to the C=C phenyl ring were stretching at 1600 cm^−1^ and the C=C stretch formed in the aliphatic portion of the PS chain, seen at 1650 cm^−1^. 

Finally, the complex region at 1300–850 cm^−1^ showed a noticeable formation of new broad bands centered at 1250 cm^−1^ due to C-O-C vibration groups and C-H bending [43,47]. This band was also related to ester contributions [48]. All the characteristic bands found in the nanocomposites were identical to those of the neat PS when exposed to UV radiation. However, the samples with 0.003 and 0.005 of 2D content showed similarities in the absorption curves for the last degradation periods, in terms of intensity, which may be related to less formation of oxidation products.

**Figure 4:**
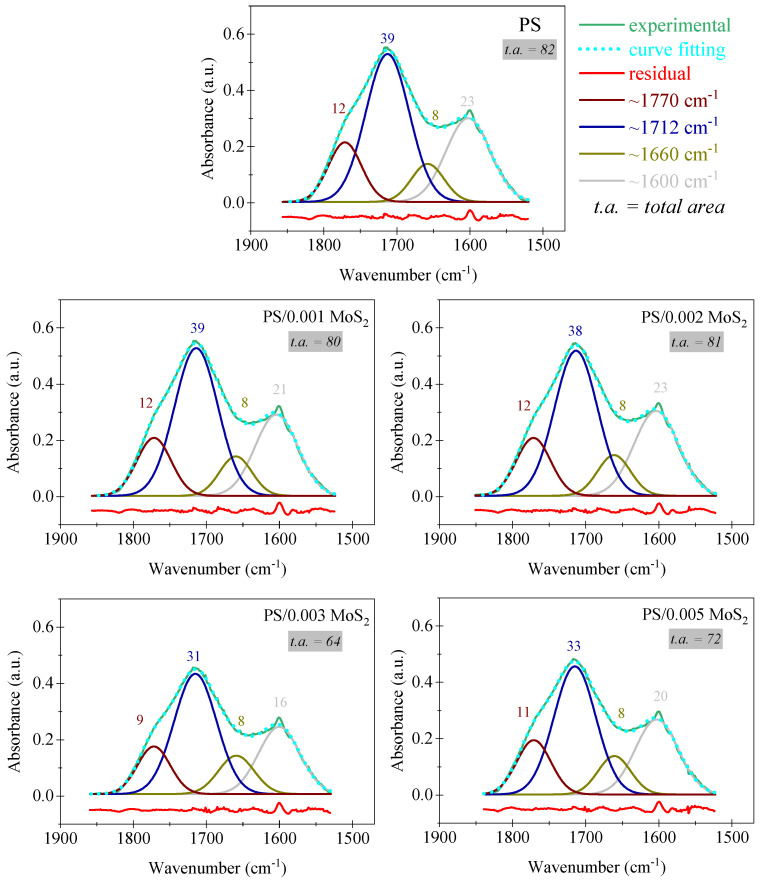
Distribution of oxidation products determined by curve-fitting of the carbonyl spectra regions samples exposed to UV radiation for 8 weeks.

An additional analysis of the carbonyl region was performed through band deconvolution using the Gaussian method. The curve-fitting method allowed the functional groups formed at the carbonyl region in absorption spectra to be distinguished. This procedure was performed only on samples exposed for 4 and 8 weeks, since the spectra for unexposed and those exposed for 1 week had a complete absence of bands and did not show significant oxidation products. As the spectra region was more defined, the deconvolution analysis of these periods provided a reasonable confidence and accuracy fit. The variation of carbonyl groups detected by curve-fitting for 8 weeks weathering samples are shown in Figure 4, as well as the normalized areas observed for each identified peak. The mathematical adjustment revealed three main bands for neat PS and nanocomposites; the different peak positions that were observed at about 1770, 1714, 1660, and 1600 cm^−1^ were assigned to isolated carboxylic acids, associated carboxylic acids, conjugated alkenes, and double bonds in the aromatic ring. The peak of greater intensity was related to the associated carboxylic acids, observed at 1712 cm^−1^. In this context, the conjugated bonds were related to the increase in double bonds, as observed in the UV-Vis spectroscopy tests. Although deconvolution at the carbonyl region did not point to ester group stretching vibrations at 1739 cm^−1^, the 1250 cm^−1^ region had high absorption (Figure 1) and was related to the C-O-C functional group [48].

A low variety of carbonyl groups and the absence of ketone groups can be seen for these periods. The last evidence may further indicate the consumption of this chemical group by degradation reactions [16]. The ultraviolet radiation was absorbed generating excited states that decompose ketone groups to other products through Norrish I mechanisms [49]. There was a variation in the carbonyl products produced; the carboxylic acid was reported to be formed under less severe aging conditions, and as the radiation becomes more intense, there is a greater formation of ester groups [50]. All those citations were observed for neat PS as well as nanocomposites, which means carbonyl and ester groups were the main products obtained here. As nanocomposites form the same oxygenated products as neat PS, it can be inferred that MoS_2_ does not interfere with pathway mechanisms. However, the identified product areas in the presence of MoS_2_ nanosheets were clearly lower. Samples with 0.003 and 0.005 filler content showed less formation of carbonyl groups; therefore, the developmental trend of these phases was not linear. In this regard, the effectiveness of PS/0.003 MoS_2_ for enhancing the photooxidation performances may be related to a better state dispersion of its nanosheets, since the photooxidation performance of composites were correlated with the degree of dispersion of fillers in the polymeric matrix [51,52].

The MoS_2_ degradation performance is due to its chemical stability and adsorptive property [23]. In the nanocomposites, the electron acceptance properties of MoS_2_ may contribute to the interfacial charge transfer of photogenerated radicals, which decreases the concentrations of species available for oxygenated products formation [21,22].

### 3.3. Molecular Weight Analysis

**Figure 5:**
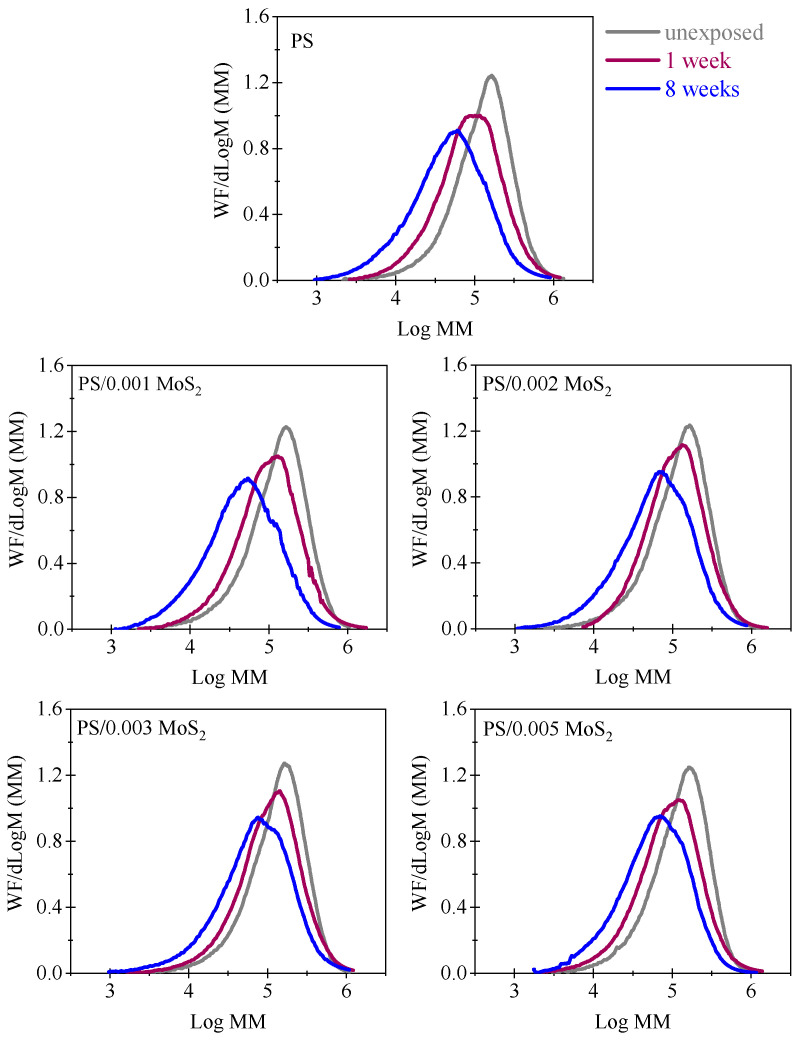
Molecular weight distribution of PS and PS/MoS_2_ nanocomposites, unexposed and exposed to UV radiation for 1 and 8 weeks.

Figure 5 shows the molecular weight curves of PS, unexposed nanocomposites, and nanocomposites exposed to up to 8 weeks of UV radiation. As the UV exposition time increased, the curves were shifted to lower molecular weight values. Table 2 presents data on the molecular weights (${\bar{M}}_{n}$ and ${\bar{M}}_{w}$) and polydispersity index $({\bar{M}}_{w}$/${\bar{M}}_{n}$) of PS and PS/MoS_2_ nanocomposites for different exposure times. The increase in the exposure time led to a decrease in the values of the average molecular weight (${\bar{M}}_{n}$) and average molecular weight (${\bar{M}}_{w}$), and an increase in the polydispersity for all samples. This behavior suggests chain scission, the most common mechanism for polymers where UV radiation is applied [31,53].

The polydispersity of unexposed samples indicates smaller values for nanocomposites with higher MoS_2_ content, and as the exposure to UV radiation increased, the ${\bar{M}}_{w}$/${\bar{M}}_{n}$ increased less for nanocomposites. 

**Figure 6:**
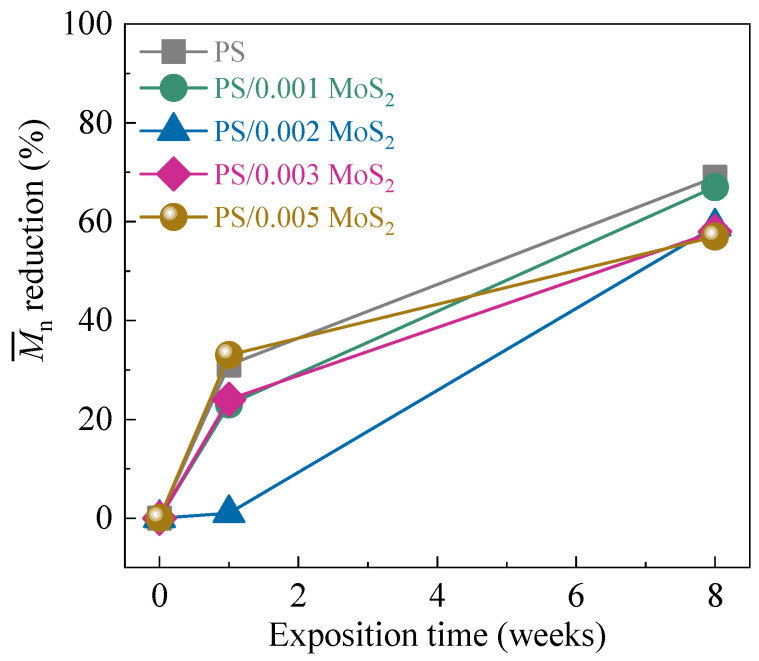
The effect of exposition time on ${\bar{M}}_{n}$.

It was evident that, after 8 weeks of UV exposition, the number of chain scissions was higher for unfilled PS and nanocomposites with lower MoS_2_ content. Figure 6 shows the ${\bar{M}}_{n}$ reduction in samples when the exposure time increased. All samples showed a significant decrease even after 1 week of UV exposition; however, after 8 weeks of exposure, unfilled PS and PS/MoS_2_ with lower MoS_2_ content showed significantly reduced values (~69%), which corroborates with the number of chain scissions data. The SEC results indicate that MoS_2_ nanoparticles can reduce the scissions reactions of PS when it is exposed to UV radiation.

PS is the material evaluated with the highest reduction in ${\bar{M}}_{n}$. After 8 weeks of accelerated aging, there was a reduction of approximately 70% in ${\bar{M}}_{n}$ and 55% in ${\bar{M}}_{w}$. For the nanocomposites, it can be observed that the compositions with higher amounts of MoS_2_ showed a smaller reduction in molar mass.

The sample with a 0.003% load showed the smallest reduction in ${\bar{M}}_{n}$ and ${\bar{M}}_{w}$ after 8 weeks of degradation; the reduction in ${\bar{M}}_{n}$ was about 11% lower than that observed for PS. This result again strongly suggests a very much increased stability of the sample, this mass decrease result corroborates with the carbonyls analysis using FTIR spectra. The 0.003% nanocomposite had fewer carbonyl groups and a smaller drop in molecular weight, indicating that this ultralow content of MoS_2_ can slow the PS photodegradation mechanism.

Based on these results, it can be said that the 2D material does not interfere with the degradation mechanism pathways of the polymer matrix since the degradation products identified are the same for neat PS. However, MoS_2_ can interfere with the deactivation of radicals. The photoinduced MoS_2_ nanosheets could scavenge the radical intermediates and, thus, would act as an antioxidant. Although MoS_2_ was at a very low level, it is noteworthy that the observed scavenge effect was due to the high surface-to-mass ratio of MoS_2_ nanosheets [21,23].

The behavior of nanocomposites in the face of accelerated degradation indicates that the deactivation of radical intermediates by MoS_2_ delayed the scissions reactions of the PS, generating fewer carbonyl groups and conjugated double bounds, as there were fewer reactive species available in the photodegradation medium. In the same way, these compositions were also sufficient to improve the mechanical properties [34].

## 4. Conclusions

The molybdenum disulfide exhibits antioxidant activity in a polystyrene-based nanocomposite under UV radiation, even considering the ultralow amount of the filler. Based on the FTIR and UV/Vis results, the PS degradation mechanism was not altered by the presence of MoS_2_ particles. However, for nanocomposites, the degradation reaction rate was decreased. The molecular weight analyses showed complementary aspects regarding chain scissions, higher concentrations samples of MoS_2_ had lower mass loss. The mathematical adjustment of the FTIR spectra distinguishes the functional groups formed during the photodegradation of PS and its nanocomposites. Among the different chemical species, carboxylic acids attracted our attention in terms of signal intensity and represents the largest population of final oxidation product. The combination of mechanical properties and photooxidative performance makes nanocomposites with ultralow content of MoS_2_, which is promising for potential high-quality material applications. At the concentrations used here, the 2D material can be considered a stabilizer in the photodegradative processes of polystyrene.

## Figures and Tables

**Table 1:** Appearance changes of unexposed and weathered PS and PS/MoS_2_ films under visible light.

	Unexposed	4 Weeks	8 Weeks
PS	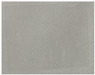	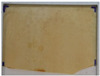	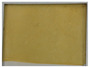
PS/0.001 MoS_2_	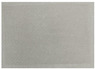	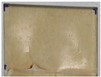	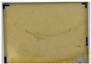
PS/0.002 MoS_2_	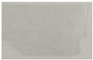	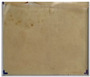	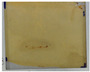
PS/0.003 MoS_2_	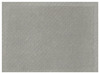	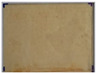	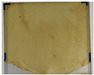
PS/0.005 MoS_2_	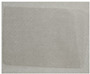	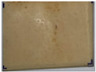	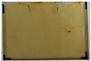

**Table 2:** ${\bar{M}}_{n}$, ${\bar{M}}_{w}$ and ${\bar{M}}_{w}/{\bar{M}}_{n}$ values of neat PS and nanocomposites, non-exposed and exposed to UV radiation.

System	Irradiation Time (Weeks)	${\bar{M}}_{n}$ (Da)	${\bar{M}}_{w}$ (Da)	${\bar{M}}_{w}$/${\bar{M}}_{n}$
PS	0	73,033	169,530	2.3
	1	50,615	127,206	2.5
	8	22,871	76,998	3.4
PS/MoS_2_ 0.001	0	72,563	168,947	2.3
	1	56,024	139,200	2.5
	8	24,156	72,920	3.0
PS/MoS_2_ 0.002	0	71,499	161,681	2.3
	1	72,247	148,225	2.1
	8	29,438	97,328	3.3
PS/MoS_2_ 0.003	0	77,421	170,730	2.2
	1	58,888	149,415	2.5
	8	32,387	109,711	3.4
PS/MoS_2_ 0.005	0	75,750	161,050	2.1
	1	51,002	131,176	2.6
	8	32,817	89,719	2.7

## Data Availability

Not applicable.
